# A comparison of total versus partial omentectomy for advanced gastric cancer in laparoscopic gastrectomy

**DOI:** 10.1186/1477-7819-12-64

**Published:** 2014-03-26

**Authors:** Dong Jin Kim, Jun Hyun Lee, Wook Kim

**Affiliations:** 1 Division of GI Surgery, Department of Surgery, Yeouido St. Mary’s Hospital, College of Medicine, The Catholic University of Korea, #62 Yeouido-dong, Yeongdeungpo-gu, Seoul 150-713, Korea; 2 Department of Surgery, Bucheon St. Mary’s Hospital, The Catholic University of Korea, #327 Sosa-dong, Wonmi-Gu, Bucheon 420-717, Korea

**Keywords:** stomach neoplasms, omentum, laparoscopy

## Abstract

**Background:**

Minimally invasive surgery has been slowly introduced into the field of advanced gastric cancer (AGC) surgery. However, the appropriate extent of omentectomy during laparoscopic gastrectomy for AGC is unknown.

**Methods:**

From July 2004 to December 2011, 146 patients with serosa-negative advanced gastric cancer were divided into the total omentectomy group (TO group, n = 80) and the partial omentectomy group (PO group, n = 66). The clinicopathologic characteristics, surgical outcomes, recurrence pattern and survival were analyzed.

**Results:**

There were no significant differences in the clinicopathologic features between the two groups, except for depth of invasion; more T3 (subserosal invasion) cases (65%) were included in total omentectomy group (*P* = 0.011). The mean time for PO was significantly shorter (35.1 ± 13.0 min) than TO (50.9 ± 15.3 min) (*P* %0.001), and there were two omentectomy-related complications in the TO group: spleen and mesocolon injuries. Recurrence occurred in 14 (17.5%) and 5 (7.6%) cases in the TO and PO group, respectively (*P* = 0.054). Disease-free survival (TO versus PO: 81.5% versus 89.3%, *P* = 0.420) and disease-specific survival (TO versus PO: 89% versus 94.7%) were not significantly different between the two groups. In the case-matched analysis using propensity score matching, there was no difference in disease-free survival (TO versus PO: 83.3% versus 90.5%, *P* = 0.442).

**Conclusions:**

Partial omentectomy might be an oncologically safe procedure during laparoscopic gastrectomy for serosa-negative advanced gastric cancer, similar to early gastric cancer.

## Background

Laparoscopic gastrectomy for advanced gastric cancer (AGC) is not widely used, but interest in the procedure is increasing [1-3]. Therefore, in Korea, the KLASS-02 trial (NCT01456598) began in 2012 to compare laparoscopic and open subtotal gastrectomy in local AGC. In the surgical procedure for AGC, D2 dissection with total omentectomy is mandatory for both laparoscopic gastrectomy and open gastrectomy. Although D2 dissection has an oncologic benefit in AGC, the role of total omentectomy is still questionable, particularly for serosa-negative AGC [4,5]. In fact, dissection through the avascular plane with proper countertraction of the transverse colon can lead to rapid and satisfactory total omentectomy during open surgery; however, in laparoscopic gastrectomy, total omentectomy is time-consuming and poses a risk of injury to the adjacent organs, particularly the spleen and colon. According to the Japanese gastric cancer treatment guidelines, partial omentectomy may be performed in cases of T1 and T2, and total omentectomy is the standard procedure for T3 or deeper tumors [6]. The main purpose of performing total omentectomy is to remove all of the potential seeding lesions in the event of serosal exposure of the tumor cells (T4a). The aim of this study was to elucidate the feasibility of partial omentectomy, based on surgical and oncologic aspects, compared with total omentectomy during laparoscopic gastrectomy for serosa-negative AGC.

## Methods

### Patients and variables

The data of 530 patients who had undergone laparoscopic gastrectomy from July 2004 to December 2011 were retrospectively reviewed. Among these patients, 146 with histologically confirmed serosa-negative AGC were evaluated. The patients were divided into a total omentectomy group (TO group, n = 80) and a partial omentectomy group (PO group, n = 66) based on the surgical procedure that they had undergone. The type of omentectomy was determined according to intraoperative gross findings regarding the status of serosal exposure. Total omentectomy was performed in cases of suspected serosal tumor infiltration, and partial omentectomy was performed in definitively serosa-negative cases. The omentectomy time was defined as the time from the initial division of the omentum to the completion of both sides of the gastroepiploic vessels. Clinicopathologic features; postoperative surgical outcomes, including the omentectomy time; disease-specific and disease-free survival; and the pattern of recurrence were compared between the two groups. To compensate for the selection bias, a case-matched analysis using propensity score matching was additionally performed based on T-stage and N-stage. This study was approved by institutional review board in Catholic Medical Center, Korea.

### Surgical procedure

All of the patients were placed in a supine position and subjected to a 15 to 20° reverse Trendelenburg position. First, routine exploration of the abdominal cavity and tumor lesion was performed to exclude peritoneal metastasis and definite serosal invasion.

Total omentectomy was performed via the same procedure as in open gastrectomy. The assistant grasped the transverse colon with an atraumatic grasper to ensure a secure dissection plane, and division of the greater omentum along the avascular plane using ultrasonic shear (Ethicon Endo-Surgery, Cincinnati, OH, USA) was started from the middle part of the transverse colon and extended up to the lower pole of the spleen. The left gastroepiploic vessels were divided to remove lymph node number 4sb. The right side of the omentum was divided along the transverse colon and the hepatic flexure. The dissection was continued toward the inferior border of the pancreas head and neck area, and the right gastroepiploic vessels were exposed and divided at their origin with removal of lymph node number 6. In cases of partial omentectomy, the division was started from the greater omentum at the line 4 to 5 cm from the gastroepiploic arcade using an ultrasonic shear toward the origin of the left gastroepiploic vessels. The omental branch was typically identified and preserved to prevent a possible omental infarct. The procedures for the right side were same as for total omentectomy.

### Statistical analysis

Clinicopathologic features and surgical outcomes were analyzed using an unpaired t-test for continuous variables and the chi-square test or Fisher’s exact test for nominal variables. In the univariate survival analysis, the Kaplan-Meier and log-rank tests were used. All statistical analyses were performed using SPSS 17.0 (SPSS Inc., Chicago, IL, USA). A *P* value of %0.05 was considered to be statistically significant.

## Results

### Clinicopathologic features and surgical outcomes

There were no significant differences between the groups, including regarding lymph node status and TNM staging, except for the depth of invasion. More T3 cases (65%) underwent total omentectomy, and more T2 cases (56.1%) underwent partial omentectomy (*P* = 0.011; Table 1). The two groups did not significantly differ regarding other clinicopathologic findings, including sex, age, the type of resection, tumor size, histologic type, resected margins, and the number of retrieved and metastatic lymph nodes. In the surgical results, the mean time for partial omentectomy was significantly shorter (35.1 ± 13.0 min) than that for total omentectomy (50.9 ± 15.3 min) (*P* %0.001). There were two omentectomy-related complications in the TO group, including spleen and mesocolon injuries, requiring concurrent splenectomy and transverse colectomy (Table 2).

**Table 1:** The clinicopathologic features of patients with serosa-negative advanced gastric cancer (AGC) according to the type of omentectomy

**Variable**		**Total omentectomy (n = 80)**	**Partial omentectomy (n = 66)**	***P***
Age (years)^a^		60.9 ± 11.2	62.2 ± 11.0	0.483
Sex	Male	56 (70)	50 (75.8)	0.438
	Female	24 (30)	16 (24.2)	
Extent of resection	Total	19 (23.8)	12 (18.2)	0.413
	Distal	61 (76.3)	54 (81.8)	
Depth of invasion	pT2	28 (35)	37 (56.1)	0.011
	pT3	52 (65)	29 (43.9)	
Lymph node metastasis	0	40 (50)	34 (51.5)	0.419
	1	14 (17.5)	8 (12.1)	
	2	13 (16.3)	16 (24.2)	
	3a	6 (7.5)	6 (9.1)	
	3b	7 (8.8)	2 (3.0)	
Tumor stage (UICC 7th)	Ib	17 (21.3)	23 (34.8)	0.130
	IIa	30 (37.5)	15 (22.7)	
	IIb	9 (11.3)	11 (16.7)	
	IIIa	13 (16.3)	12 (18.2)	
	IIIb	11 (13.8)	5 (7.6)	
Tumor size (cm)^a^		4.8 ± 2.5	4.4 ± 2.4	0.366
Histologic type	Differentiated	37 (46.3)	24 (36.4)	0.228
	Undifferentiated	43 (53.8)	42 (63.6)	
Proximal margin (cm)^a^		4.0 ± 2.1	3.7 ± 2.2	0.357
Distal margin (cm)^a^		5.3 ± 3.4	6.4 ± 4.2	0.083
Lymphatic invasion	Absent	27 (33.8)	30 (45.5)	0.149
	Present	53 (66.3)	36 (54.5)	
Perineural invasion	Absent	39 (48.8)	40 (60.6)	0.152
	Present	42 (51.3)	26 (39.4)	

^a^Mean ± standard deviation; nominal variables are expressed as number (%).

**Table 2:** Surgical outcomes in patients with serosa-negative advanced gastric cancer (AGC) according to the type of omentectomy

**Variable**		**Total omentectomy (n = 80)**	**Partial omentectomy (n = 66)**	***P***
Lymph node (LN) dissection	D1 + β	2 (2.5)	6 (7.6)	0.153
	D2	78 (97.5)	61 (92.4)	
Number of retrieved LNs^a^		34.6 ± 14.7	39.8 ± 14.7	0.034
Number of metastatic LNs^a^		4.3 ± 8.5	2.9 ± 5.3	0.228
Omentectomy time (min)^a^		50.9 ± 15.5	35.1 ± 13.0	%0.001
Omentectomy-related complication		2^b^	0	0.198
Recurrence		14 (17.5)	5 (7.6)	0.076

^a^Mean ± standard deviation; nominal variables are expressed as number (%).

^**b**^Complications included intra-operative spleen and mesocolon injury, requiring concurrent splenectomy and transverse colectomy.

### Recurrence and survival

During the follow-up period, a total of 19 recurrences were identified, including 14 (17.3%) in the TO group and 5 (7.6%) in the PO group. Among the T2 cases, 2 recurrences occurred in the 3rd-tier lymph node and bone in the TO group, and 2 recurrences occurred in the bone, with simultaneous 3rd-tier lymph node metastasis and remnant stomach cancer in the PO group. Among the T3 cases, there were 13 recurrences in the TO group: 3 carcinomatoses, 3 distant lymph node metastases, 3 remnant stomach tumors, and 4 cases of hematogenous spread (3 in the liver and 1 in the bone). Three recurrence cases, including 1 carcinomatosis, 1 liver site, and 1 port site, occurred in the PO group among T3 cases (Table 3).

**Table 3:** Recurrence pattern according to the type of omentectomy and the depth of invasion

**Depth (n)**	**Omentectomy (n)**	**Recurrence (n)**	**Site (n)**
pT2 (65)	Total omentectomy (28)	2	3rd-tier lymph node (LN) (1)
			Bone (1)
	Partial omentectomy (37)	2	3rd-tier LN + bone (1)
			Remnant stomach (1)
pT3 (81)	Total omentectomy (52)	12	Carcinomatosis (3)^a^
			Liver (3)
			Bone (1)
			3rd-tier lymph node (2)
			Remnant stomach (3)
	Partial omentectomy (29)	3	Carcinomatosis (1)^a^
			Liver (1)
			Port site (1)

^a^Proportion of carcinomatosis in total omentectomy versus partial omentectomy: 3/80 (3.8%) versus 1/66 (1.5%), *P* = 0.410 (chi-square analysis).

**Figure 1:**
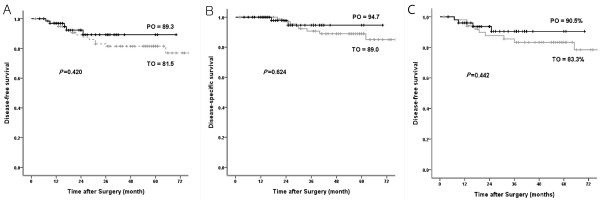
**Survival analysis. (A)** Disease-free survival did not differ between the total omentectomy (TO) and the partial omentectomy (PO) groups. **(B)** Disease-specific overall survival did not differ between the total omentectomy and the partial omentectomy groups. **(C)** Disease-free survival did not differ between the matched groups.

There were no significant differences in cumulative disease-free survival (TO versus PO: 81.5% versus 89.3%, *P* = 0.420) or disease-specific survival (TO versus PO: 89.0% versus 94.7%, *P* = 0.624) between the two groups (Figure 1). In addition, there was no difference in the development of recurrence in the omentum or of carcinomatosis (TO versus PO: 3/80 (3.8%) versus 1/66 (1.5%), *P* = 0.410) (Table 3). To overcome the selection bias due to the T-stage discrepancy between the two groups, Cox proportional analysis was performed for recurrence with the following covariates: the type of omentectomy, tumor depth, and lymph node status (Table 4). This analysis revealed that the type of omentectomy was not a risk factor for recurrence.

**Table 4:** Univariate and multivariate analyses of the risk factors for recurrence

**Variable**		**Univariate**		**Multivariate**	
		**5 yr DFS**^**a**^** (%)**	***P***	**Odds ratio**	***P***
Type of omentectomy	Total	81.5	0.42	1	0.766
	Partial	89.3		0.85 (0.30 to 2.43)	
Depth of invasion	pT2	92.5	0.015	1	0.082
	pT3	77.5		3.00 (0.87 to 10.39)	
Lymph node metastasis	Absent	97.2	%0.001	1	0.003
	Present	68.5		9.98 (2.06 to 39.14)	

^a^Disease-free survival.

### Case-matched analysis

Propensity score matching yielded 51 patients in each group. Table 5 shows the same proportions of T-stage and N-stage in the two matched groups. A comparison of disease-free survival between the two matched groups also showed no difference (TO versus PO: 83.3% versus 90.5%, *P* = 0.442) (Figure 1C).

**Table 5:** Proportions of T-stage and N-stage in matched groups

**Variable**		**Total omentectomy (n = 51)**	**Partial omentectomy (n = 51)**	***P***
Depth of invasion	pT2	22 (43.1)	22 (43.1)	1.000
	pT3	29 (56.9)	29 (43.9)	
Lymph node metastasis	0	28 (54.9)	28 (54.9)	1.000
	1	8 (15.7)	8 (15.7)	
	2	11 (21.6)	11 (21.6)	
	3a	2 (3.9)	2 (3.9)	
	3b	2 (3.9)	2 (3.9)	

## Discussion

Laparoscopic gastrectomy for early gastric cancer (EGC) has been widely performed, and its advantages over open gastrectomy have been verified in many randomized clinical trials [7-11]. Recently, laparoscopic gastrectomy for AGC has been increasingly performed, and certain reports have outlined its feasibility from technical and oncologic perspectives [1-3]. In laparoscopic gastrectomy for AGC, D2 dissection and total omentectomy have been the primary troublesome issues for many laparoscopic surgeons.

Although the greater omentum is known to play a role in peritoneal defense by adhering to sites of inflammation and absorbing bacteria and other contaminants, it is a common site of both recurrent disease and primary seeding in gastrointestinal malignancies [12]. Additionally, several experimental studies have reported that cancer cells seeded in the peritoneal cavity preferentially grow on the omentum [12,13]. For this reason, total omentectomy has been a standard operative procedure during open gastrectomy, regardless of tumor depth. However, if there is no serosal exposure, cancer cell spillage or spread through the omentum is not theoretically possible. Therefore, we hypothesized that partial omentectomy is sufficient for T2 and even T3 cases. The most important type of recurrence relevant to omentectomy may be carcinomatosis. Kim *et al.* and Ha *et al.* reported no survival difference between TO and PO in EGC [14,15]. Kim *et al.*[16] reported no difference in the pattern of recurrence between TO and PO in AGC without serosal exposure during open gastrectomy. The researchers also detected no difference in the rate of peritoneal metastasis among all recurrences (35% and 25% in TO and PO, respectively). In our series, although more recurrences were noted in the TO group than in the PO group, there was no difference in survival between the two groups. In addition, there was no difference in the occurrence of carcinomatosis between the two groups. Interestingly, approximately half of the T3 cases (25 cases) in the TO group were regarded as including definite serosal invasion (T4a) intra-operatively but were finally proven to encompass subserosal invasion (T3) histologically. Despite the paucity of serosal exposure in the pathologic findings, certain investigators believe that the peritoneum can form a new surface over exposed cancer cells in clinically T4a lesions. Koji *et al.* reported that the width of subserosal invasion is an independent risk factor for survival in histologically confirmed T3 gastric cancer [17]. We believe that clinical serosal exposure in histologically confirmed T3 cases involves a larger width of subserosal invasion or even focal serosal penetration, which may be why the number of recurrences was much higher in the TO group.

Regarding the high incidence of remnant gastric cancer, we were unable to determine the exact reason because all of the cases had negative margins for malignancy and because remnant gastric cancer lesions were not involved in the anastomosis line. The Japanese gastric cancer treatment guidelines (2010; version 3) recommend leaving a proximal margin of at least 3 cm in the presence of an expansive growth pattern and of 5 cm in the presence of an infiltrative growth pattern or evaluating frozen sections when these factors cannot be observed [6]. Considering that the interval between the initial operation and the completion of gastrectomy was relatively short (1 year for 2 patients and 2 years for the others), there may have been undetected cancer lesions in the remnant stomach, despite preoperative gastrofiberscopy.

Moriguchi *et al.*[18] have reported that serosal invasion and Borrmann type 4 carcinoma are independent risk factors for the development of carcinomatosis. Although no randomized controlled trials have been performed, Fugita *et al.*[19] also showed that the type of omentectomy was not a risk factor for recurrence in serosa-negative AGC in a retrospective study, and they included several serosal exposure cases (T4a). The researchers also showed that the development of carcinomatosis has no relationship with the type of omentectomy. The most important factor for justifying the selection of partial omentectomy is tumor depth. The accuracy of preoperative evaluation by endoscopic ultrasonography (EUS) was reported to be 85.7%, and Kim *et al.* reported that the accuracy of macroscopic findings in determining whether a tumor had invaded the serosa was 87% [16]. Therefore, if we select the type of operation conservatively, with consideration of preoperative evaluations, we can avoid performing partial omentectomy in T4a cases.

In previous reports comparing total omentectomy and partial omentectomy in EGC, partial omentectomy showed several advantages over total omentectomy, including in operation time, perioperative complications, and the postoperative albumin level [14,15]. Total omentectomy in open gastrectomy is no more difficult than partial omentectomy. With traction of the transverse colon by an assistant, the dissection of the greater omentum can be easily performed through the avascular plane. Otherwise, in laparoscopic gastrectomy, total omentectomy can be a more challenging procedure because maintaining the dissection line through the avascular plane and dividing the omental tissue from the mesocolon are not easy, particularly in patients with a high BMI. In the present study, PO demonstrated several advantages in terms of surgical outcomes. The omentectomy time was shorter, and omentectomy-related complications did not occur in the PO group. However, omental infarction may occur during PO and can appear as carcinomatosis or omental recurrence in radiologic findings [3]. It is important to differentiate between various radiological findings and omental infarcts [20], and close follow-up is required when differentiation is difficult, particularly in the immediate postoperative period.

Because the present study was retrospectively designed, it has certain limitations. Although there was no significant difference in the distribution of stages between the two groups, the TO group contained more advanced cases. These discrepancies in tumor staging may influence the recurrence rate. However, omentectomy was not risk factor for recurrence in the multivariate analysis. Despite these limitations, the present study is valuable because previous reports were based on open gastrectomy, so this is the first report to evaluate the role of the type of omentectomy in laparoscopic gastrectomy for serosa-negative AGC.

## Conclusions

In conclusion, partial omentectomy can be a useful alternative method for performing laparoscopic gastrectomy for serosa-negative AGC. However, to determine long-term technical and oncologic safety, a prospective randomized controlled trial is needed.

## Abbreviations

AGC: advanced gastric cancer; BMI: body mass index; EGC: early gastric cancer; EUS: endoscopic ultrasonography LN, lymph node; PO: partial omentectomy; TO: total omentectomy.

## Competing interests

All authors declare that they have no competing interests.

## Authors’ contributions

WK performed the surgeries and organized the entire study. DJK drafted and wrote this manuscript. JHL participated in the study design and revised the manuscript. All authors read and approved the final manuscript.
